# Cumulative family risks across income levels predict deterioration of children’s general health during childhood and adolescence

**DOI:** 10.1371/journal.pone.0177531

**Published:** 2017-05-16

**Authors:** Yi-Ching Lin, Dong-Chul Seo

**Affiliations:** 1 Department of Early Childhood and Family Education, College of Education, National Taipei University of Education, Taipei, Taiwan; 2 Department of Applied Health Science, Indiana University School of Public Health, Bloomington, Indiana, United States of America; Royal Children's Hospital, AUSTRALIA

## Abstract

Family is considered an important agent in the health development of children. This process is significant but quite complex because the prevalence of potential risk factors in the family can hinder children’s health. This study examined if multiple family risks might have cumulative effect on children and youth’s health across various levels of household income. The data in this study were drawn from the 2011–2012 U.S. National Survey of Children’s Health (N = 79,601). A cumulative family risk (CFR) index was developed, which included such constructs as single-parenthood, unstable employment, large family, parenting stress, poor maternal education, poor maternal general health and poor maternal mental health. Multiple logistic regression analyses showed that CFR level was significantly related to children and youth’s poor health outcome (*p* < .001). When poverty levels were considered, however, the impact of CFRs on children and youth’s health was attenuated. The impact of CFRs was higher on children and youth from affluent families than on those from poor families. Overall there was a consistent pattern of trend in the point estimate as well as confidence limits as levels of affluence and numbers of family risk increased although some of the confidence intervals overlapped. Living in disadvantaged families might serve as a protective factor against CFRs possibly through repeated exposure to hardships and subsequent formation of resilience among some of the disadvantaged children.

## Introduction

Family is considered an important agent in the health development of children[1]. It plays a large role in how children learn and grow throughout their childhood and adolescence [2, 3]. This process is significant but quite complex because the prevalence of potential risk factors in the family can hinder the health and development for children and youth [4, 5]. Risk can refer to any individual, social, or environmental factor that leads to undesirable or adverse development of children [6]. However, individual risk factors do not occur in a vacuum and often cluster together. Multiple family risks may concurrently and accumulatively affect children and youth, jeopardizing their development and health [4]. Moreover, cumulative family risk factors may invoke children and youth’s vulnerabilities [7, 8], because managing accumulated risks is extremely demanding for them and can induce distress and compromise their normative development [4]. Such distress can be overwhelming, adverse, and harmful to the children and youth’s general health [9, 10], mental health [11], behavior [12], school performance [13], psychosocial ability [14], and adjustment capability [4].

Cumulative risk approaches provide methods to examine how risk factors function, interact, and shape the overall health of children [15]. The advantage of using aggregated scores, in contrast to using a single risk factor, is the possibility of simultaneously measuring all accumulated risks within a particular family domain, which enables researchers to compare risk levels across groups. In addition, several studies have suggested that cumulative risk aggregations show stronger correlation with individual outcomes than do any single risk factor [15, 16]. These aggregated scores are presented in a cumulative risks index. The index comprises several dichotomized risk factors such as maternal illness, maternal anxiety, low maternal education, parenting stress, overcrowded housing, stressful life event, and job insecurity [9, 16, 17]. An increase in number of coexisting risk factors with long-standing adversities has been found to be injurious and destructive to the later developmental outcomes of children [15, 18].

The findings of the Rochester Longitudinal Study show that children in high-risk groups (i.e., 8 or more risk factors) were approximately 7 times more likely to have poor academic outcomes than were children in low-risk groups (i.e., 0–3 risk factors) [13]. Another study indicated that children with more than 6 risk factors were 17.31 times more likely to be of poorer health compared with those with no risk factors [19]. Although studies have demonstrated strong associations between cumulative risks and children’s health, little is known about the impact of poverty, as a social context, on children’s health outcomes [20]. Poverty is social status that contains a set of interrelated contexts, circumstances and experiences, such as divorced families, poor education, and being in a minority group. In turn, such characteristics affect the context surrounding the children within the home [21]. Through the perspective of ecological system model [22], it is assumed that a distal risk factor such as poverty may not directly impact child’s health but rather may influence it indirectly through other factors that are more proximal to the child [23].Therefore, the contextual influence should be examined through ecological system perspective to identify specific proximal and distal factors that place children with cumulative family risks on their health.

Cumulative family risks (CFRs) capture the scope of ecological covariation that are exposed to risks by developing a measure that simultaneously accesses multiple sources of risk [16]. Eight risk factors that have been identified to predispose children to suboptimal health are under consideration of the current study: poverty [16, 24], single parenthood [25, 26], family stress [27–29], unstable employment [30, 31], large families [32, 33], poor maternal health [34], emotional health[35–37] and education [38, 39]. They can be managed as two segments: distal and proximal. From an ecological perspective [22], capturing effects of distal indicators is important as it adds contextual understanding to the relations between proximal indices of risk and child health quality outcomes. This is a theoretically compelling approach to risk research [16, 40] because ecological approaches provide structural framework especially when it is difficult to disentangle the effect of poverty per se and risk factors common in disadvantaged families [41, 42]. Furthermore, the framework of social determinants suggests that poor health of children and barriers to their development are largely caused by family poverty [43]. Poverty is generally conceptualized as the lack of financial resources to solve problems and make life choices, the prevalence of material difficulties and contextual barriers, and low accessibility to available resources [43–46]. More importantly, poverty often leads to impaired parenting, family conflict, and poor health outcomes for children [47, 48], which underscores the potential impact of poverty on the association of CFRs and child health. There is also reasonable evidence of linear relations between singular risk factors and poverty. However, little is known about exposure to multiple risks across varying levels of poverty[49, 50].

In addition, contextual factors, both proximal and distal, profoundly affect the outcome of children’s health [16], but how the effects interact with the outcome has received limited attention. Children with high CFRs, which are a proximal factor, may have long suffered from a series of adversities and social disadvantages. In other words, the association between children’s health risk and CFR could be moderated by poverty level. Hence, the question is, “Could the situation deteriorate with additional negative distal impact?” The current study attempts to answer this question by examining the impact of CFRs across poverty levels on child health [51].

## Method

### Participants

The study data were retrieved from the 2011–2012 U.S. National Survey of Children’s Health, which was sponsored by the Maternal and Child Health Bureau of the U.S. Health Resources and Services Administration. The survey was conducted as a random-digital-dial survey and the results have been weighted to represent the population of non-institutionalized children aged 0–17 at both nationwide- and state- levels [52]. The respondent was a parent or guardian in the household who was knowledgeable about the child’s health [53].

The data includes information regarding the states of children’s physical, emotional health as well as their wellbeing, such as family functions, parental health, medical homes, insurance information, and safe neighborhoods. In this study, the inclusion criterion for the survey respondents was providing complete responses for all the family risk indicators, poverty levels, and covariate variables. The inclusion criterion yielded a valid sample size of 79,601 cases. The de-identified NSCH data are publicly available from is available from http://www.cdc.gov/nchs/slaits/nsch.htm and the approval from the institutional review board was exempted. [54]

### Measures

#### CFR index

The following indicators constituted the CFR Index and were dichotomized as described herein:

(1) *Single-parenthood* that was defined as a single parent who may be separated, divorced, or widowed was coded “1” while families with two parents were coded “0”; (2) *unstable employment* defined as families with no member employed for at least 50 of the past 52 weeks was coded “1” while others were coded “0”; (3) *large family* that was defined as a family with four or more children was coded “1” while others were coded “0”; (4) *parenting stress* was coded “1” if parents answered “coping somewhat well”, “not very well” or “not very well at all” to the question, “In general, how well do you feel you are coping with the day to day demands of parenthood/ raising children?” whereas having parents who answered “very well” were coded “0”; (5) *poor maternal education* level was defined as mothers whose highest education level was a high school degree or lower and coded “1” whereas others were coded “0.” The data of (6) *poor maternal general health* and (7) *poor maternal emotional health* were retrieved through two questions, “Would you say that, in general, your health (and emotional health, respectively) is excellent, very good, good, fair or poor?” The responses were dichotomized by combining the self-reported responses. Excellent and very good were combined as having “good health” (coded 0) and good, fair, and poor were combined as having “poor health” (coded 1).

The cumulative risks exposure (0–7) was calculated by summing the 7 single risk indicators, following which the score was categorized into 5 levels: children exposed to 0, 1, 2, 3, 4 or more risk indicators. Groups with 4 or more risk indicators were combined for analyses because the small sample size of respondents with 5 and 6 risks.

#### Poverty levels

Poverty levels were defined in accordance with the Federal Poverty Guidelines of the Department of Health and Human Services. This variable consisted of 4 levels: 1) below 100% of the Federal Poverty Level (FPL), 2) 100%–199% of the FPL, 3) 200%–399% of the FPL, and 4) at or above 400% of the FPL.

#### Children’s general health

The health outcome for children in this study was parent-reported child health status. In the survey, parents were asked by the question: “In general, how well do you describe your child’s health? Would you say his/her health is excellent, very good, good, fair, or poor?” This variable was dichotomized by combining the responses of excellent and very good (as one category labeled as good health), and those of good, fair, and poor (as the other category labeled as poor health). Studies have shown that adults tend to overestimate health condition, internalizing problems, or physical activity for themselves or for their children [55–57]. Thus, we collapsed responses of “good” with responses of “fair” and “poor.”

### Statistical analyses

This research had two objectives: to determine 1) whether CFR and children’s health were associated for the current population, and 2) whether the association changes with poverty levels. Statistical significance was calculated through bivariate associations between the variables, which were examined using cross tabulations and *X*^2^ tests. In addition, multivariable logistic regression was performed to examine the associations among CFRs, poverty levels, and children’s general health with 95% confident interval after controlling for age, sex, and ethnicity. The interaction between CFRs and poverty level was first tested to validate the effect of the former on children’s health across poverty levels. Family income of 400%+ of the FPL and zero family risks were used as reference categories. In each of the income levels, a significant gradient was used to indicate the extent to which the cumulative family risk deteriorated children and youth health.

## Results

The sample consisted of 79,601 children and youth aged 0–17 years. The average age was 8.75 years (SD: 5.25); 51.2% of the sample were boys and 67% were non-Hispanic White. Approximately one third of the participants (31.7%) were from low-income families below the poverty line (200% FPL). The prevalence of poor health for each family risk indicator and the accumulated family risk level is listed in Table 1. Approximately 40% of the families reported to not be coping with family stress well, and approximately 30% of the mothers with low levels of education or poor health (emotional or overall health). In addition, about 7% of the participants come from large families having 4 more children living in a household. More than 60% lived with no or 1 family risk, whereas 7.4% lived with 4 or more family risks. Pearson Chi-square tests were used to compare the demographic categories and family risk indicators in the children and youth’s health groups. As shown in Tables Tables 1 and 2, all results were statistically significant for every category at the p < .001 level.

**Table 1:** Descriptive statistics for sample background and risk indicators, NSCH 2011–2012 (N = 79,601).

Variables	Description	Total	Prevalence of poor health		
		%	%	*p*-value	chi-square
**Sex**				< .001	44.31
	Boys	51.2	12.1		
	Girls	48.8	10.6		
**Race/ethnicity**				< .001	2522.97
	Hispanic	13.3	24.3		
	White, non-Hispanic	67.0	8.0		
	Black, non-Hispanic	8.7	16.0		
	Multi-racial/Other, non-Hispanic	11.0	12.6		
**Poverty levels**				< .001	3385.96
	At or Below 200% of poverty level	31.7	20.8		
	Above 200% to at or below 400% of poverty level	30.9	8.8		
	Above 400% of poverty level	37.4	5.5		
**Family risk indicators**					
Single Parenthood	Single-parent family and other kind	17.3	27.5		
Parenting Stress	Coping parenting stress somewhat well/not very well/not very well at all	39.1	49.1		
Unstable Employment	No one in the household employed at least 50 weeks out of the past 52 weeks	10.9	21.9		
Large Family	With 4 or more children living in a household	6.8	8.9		
Poor Maternal Health	Good/Fair/Poor health condition	31.3	63.8		
Poor Maternal Education	High school graduated and less than high school	24.6	51.4		
Poor Maternal Emotional Health	Good/Fair/Poor emotional health condition	25.5	47.1		
**Counts of Cumulative Family Risk Scale**					
0	Had 0 family risk indicator	30.3			
1	Had 1 family risk indicator	30.6			
2	Had 2 family risk indicators	18.8			
3	Had 3 family risk indicators	12.8			
4 or more	Had 4 or more family risk indicators	7.4			

**Table 2:** Individual family risk indicator to children’s poor health.

	Odds Ratio	95% Confidence Interval	
Large family	1.34***	1.24	1.45
Single-parenthood	1.69***	1.60	1.78
Parenting stress	1.58***	1.51	1.66
Unstable employment	2.22***	2.09	2.35
Poor maternal education	2.47***	2.36	2.59
Poor maternal emotional health	3.55***	3.39	3.72
Poor maternal general health	4.13***	3.94	4.33

*Note*.

*** *p* < .001

Single Parenthood: Single-parent family and other kind

Parenting Stress: Coping parenting stress somewhat well/not very well/not very well at all

Unstable Employment: No one in the household employed at least 50 weeks out of the past 52 weeks

Large Family: With 4 or more children living in a household

Poor Maternal Health: Good/Fair/Poor health condition

Poor Maternal Education: High school graduated and less than high school

Poor Maternal Emotional Health: Good/Fair/Poor emotional health condition

Bivariate logistic regression was performed to examine the association between individual family risk indicators and participants’ general health. The results revealed that each family risk indicator was significantly associated with children and youth’s general health outcome. Participants living in large families (OR = 1.34, 95% CI = 1.24–1.45) and non-intact families (OR = 1.69, 95% CI = 1.60–1.78) were more likely to be of poor health than their counterparts. Furthermore, parenting stress increased the risk of poor health (OR = 1.58, 95% CI = 1.51–1.66). Participants of parents without stable employment were more likely (OR = 2.22, 95% CI = 2.09–2.35) to have poorer health than were those of parents with stable employment. Regarding maternal risk indicators, children and youth of mothers with low levels of education, emotional, and general health were, respectively, more likely to have poorer health, as shown in Table 2.

The 7 indicators were summed to generate a CFR index by categorizing the number of family risks into 5 levels: 0, 1, 2, 3, and 4 or more risks. Subsequently, logistic regression (controlled for sex, age, and ethnicity) was conducted to investigate the relationship between CFRs and children and youth’s poor health. Table 3 shows that each CFR level was significantly related to their poor health outcome. A gradient clarified the deteriorating impact on children and youth’s health as the number of family risks accumulated. The increases in odds with each additional family risk were significant (all *p*s < .001)

**Table 3:** Cumulative family risk, poverty levels and children's poor health.

	Unadjusted Model			Adjusted Model		
	COR	95% Confidence Interval		AOR	95% Confidence Interval	
**Sex (ref: female)**	0.86***	0.82	0.90	0.85***	0.82	0.90
**Age**	1.03***	1.02	1.03	1.03***	1.02	1.03
**Ethnicity (ref: Hispanic)**						
Non-Hispanic, White	0.27***	0.26	0.29	0.44***	0.41	0.46
Non-Hispanic, Black	0.59***	0.55	0.64	0.67***	0.62	0.73
Non-Hispanic, Multi-racial/Other	0.45***	0.42	0.49	0.62***	0.57	0.67
**Federal Poverty Level** (ref: Above 400% FPL)						
Below 200% FPL	4.48***	4.22	4.74	2.70***	2.27	3.22
200%-400% FPL	1.64***	1.54	1.75	1.38***	1.18	1.62
**Family Risk (ref: 0)**						
Family Risk = 1	2.00***	1.84	2.17	1.83***	1.59	2.10
Family Risk = 2	4.07***	3.75	4.42	3.65***	3.15	4.24
Family Risk = 3	8.15***	7.51	8.84	6.60***	5.63	7.74
Family Risk ≧4	15.07***	13.83	16.43	8.96***	6.70	11.99

*Note*.

*** *p* < .001

When poverty levels were considered, however, the impact of CFRs on children and youth’s health attenuated (Table 3). The tendency of having poor health increased from 1.83 (95% CI: 1.59–2.10) (1 risk) to 3.65 (95% CI: 3.15–4.24) (2 risks), 6.60 (95% CI: 5.63–7.74) (3 risks), and 8.96 (95% CI: 6.70–11.99) (4 or more risks) (*p* < .0001). It appears that poverty modified the association between CFRs and child health. We therefore tested the interaction between CFRs and poverty levels and it was significant at the .05 level. When the interaction effect was probed through simple effects analysis, it was found that while participants from poor families and with higher family risks were associated with poor health, the difference in the outcome narrowed as income level increased. This indicates that children and youth from affluent families might be more vulnerable to the influence of cumulative family risk than their counterparts. According to these findings (Table 3), the impact of CFRs across poverty levels was explored further.

Table 4 presents the odds of children having poor health at each CFRs level across the identified poverty levels. For children and youth living in households below poverty line (<200% FPL), the odds of poor health increased from 1.44 (95% CI: 1.23–1.70) (1 risk), to 7.17 (95% CI: 6.17–8.33) (4 or more risks). However, the odds of poor health of those from households at or above 400% FPL increased from 1.86 (95% CI: 1.62–2.14) (1 risk) to 9.49 (95% CI: 7.09–12.69) (4 or more risks). Table 4 also shows the gradient of the odds that are prominent at each income level. More importantly, as the household income increased, the discrepancy between the odds of being in poor health for cases with no CFRs and for high number of CFRs became larger. Thus, the findings suggest that children and youth from affluent families could be more vulnerable to CFRs than are those from disadvantaged families.

**Table 4:** Comparison of the prevalence and the odds of poor health by cumulative family across poverty levels.

	<200%FPL		200%-400%FPL		>400%FPL		Total
	n	OR	n	OR	n	OR	n
	(%)	95% CI	(%)	95% CI	(%)	95% CI	(%)
Family Risk = 0	221	1	288	1	359	1	868
(ref.)	(4.2%)		(13.3%)		(21.8%)		(9.6%)
Family Risk = 1	642	1.44***	548	1.82***	501	1.86***	1691
	(12.2%)	(1.23, 1.70)	(25.4%)	(1.57, 2.10)	(30.4%)	(1.62, 2.14)	(18.7%)
Family Risk = 2	1041	2.44***	546	3.19***	392	3.83***	1979
	(19.9%)	(2.09, 2.84)	(25.3%)	(2.75, 3.70)	(23.8%)	(3.31, 4.45)	(21.9%)
Family Risk = 3	1535	4.59***	517	5.73***	328	6.89***	2380
	(29.3%)	(3.95, 5.33)	(23.9%)	(4.92, 6.67)	(19.9%)	(5.88, 8.08)	(26.3%)
Family Risk **≧**4	1802	7.17***	261	9.65***	67	9.49***	2130
	(34.4%)	(6.17, 8.33)	(12.1%)	(8.01, 11.63)	(4.1%)	(7.09, 12.69)	(23.5%)
**Total**	5241	—	2160	—	1647	—	9048

*Note*. FPL: Federal Poverty Guideline. ref. = reference group

*** *p* < .001

Overall there was a consistent pattern of trend in the point estimate as well as confidence limits as levels of affluence and numbers of family risk increased although some of the confidence intervals overlapped. However, there was no statistical difference in the odds ratios between CFR3 and CFR4+ for the >400% FTL group, which means that for the participants in the most affluent families, the odds difference between CFR3 and CFR4+ did not reach statistical significance. The statistical differences of the set of odds ratios was tested using Z values and the result confirmed the statistical insignificance as well.

All analyses were conducted using the Statistical Package for Social Sciences statistical software version 20.0 (IBM Corp., Armonk, NY).

## Discussion

The findings of this study indicated that each investigated family risk, especially risks related to maternal condition, was significantly associated with the suboptimal health of children and youth. The accumulation of family risks corresponded to the rise in the poor health of them. These findings are consistent with previous studies [4, 9, 19, 58]. Our analyses showed that the odds of deterioration increased, as demonstrated by a steep gradient. This implies that the accumulation of family risk factors drastically increased the threat for children and youth’s health. However, when poverty levels were considered, the impact of CFRs on their health was attenuated. This was due to the interaction between poverty levels and CFRs. The impact of CFRs was higher on children and youth from affluent families than on those from poor families. One possible speculation is that children and youth from affluent families may be exposed to less hardship and thus may not be able to cope with CFRs better than the ones from poor families. Also, there is potential for parents in different socioeconomic circumstances to rate their children’s health differently. For instance, parents who have greater economic resources or higher educational background in difficult circumstances may be more likely to identify issues affecting their children’s health and rate their children’s health as less than very good, compared to parents with few economic resources or lower education in the same circumstances.

As mentioned earlier, research studies have claimed that CFRs aggravate child health and well-being and that poverty is a predominant threat that exacerbates the negative impact on child health [59–63]. On the contrary, this study finds that living in disadvantaged families might serve as a protective factor against CFRs possibly through repeated exposure to hardships and subsequent formation of resilience among some of the disadvantaged children.

The American Psychological Association [64] defines resilience as “the process of adapting well in the face of adversity, trauma, tragedy, threats, or even significant sources of stress (paragraph 4)”. In the family unit, resilience refers to the abilities of an individual or a family to achieve specific objectives despite the challenges and risks that can disrupt their health and wellness [44, 46, 65–69]. The term has an intrinsic sense of optimism and plasticity, such as the abilities to recover from overwhelming pressure and be flexible to adapt to ongoing challenges.

Resilience at the levels of family and community was deemed essential to being resourceful in order to enable positive health outcomes [70]. It is also a process through which families adapt and function after traumatic incidents or negative experiences such as poverty [71]. Adversity in circumstances stimulates children’s sensitivity that shapes their brain development and plasticity [68]. Children from low-income households may experience more stress and conflict because of their poor living conditions and limited life choices and resources [65]. However, they may still be able to overcome these challenges through their resilience, which is a capacity to adapt to situations that threaten their functions, viability, or development [72] in order to survive [73, 74].

Therefore, competence-based programs that target capability and strength development for prevention or intervention deserve equal attention as do deficit-based studies [7, 67, 75, 76]. This perspective has profound implications for health support services, preventions, and interventions that maximize optimal responses to challenges and adversities faced by families, especially those at a disadvantage, under cumulative risks.

This study has limitations. First, the data were cross-sectional and thus no causal inferences could be derived. Future studies may be able to verify directional causality between the variables if longitudinal data can be accessed and analyzed. Second, child health was self-rated by parents, which could have confounded the results because parents tend to overestimate their child’s health [77, 78]. We tried to attune the potential bias by taking into account such parent’s tendency of overestimation of their child’s health on the cutoff of dichotomization of the responses. Third, the equal treatment of all risk factors (i.e., each is assigned a value of 1) could have biased the results because in reality some may have a bigger effect on health outcomes than others, let alone possible interaction effects amongst them.

Despite these shortcomings, this study demonstrated the impact of CFRs on children and youth’s health by integrating ecological perspectives and analyzing the influences of poverty. Several studies have discussed cumulative risks in a familial setting but very few, if any, have assessed its impact on child health based on stratified income levels. This study therefore contributes to the literature by recognizing that poverty could make children resilient in the face of adversity and motivate them to pursue health. Our results may differ from studies that claim that poverty would always be detrimental to children’s health, but challenging this understanding provides a novel phenomenon to address and problems to redefine.

## Conclusion

This study elucidates that family risks, single or cumulative, aggravate children and youth’s health. The ones who exposed to larger numbers of family risks should be prioritized for intervention. Interestingly, however, our findings corroborate that adversity may actually encourage plasticity in children from disadvantaged families. The poor who suffered from relatively higher economic hardship and family challenges were the most vulnerable, but they exhibited a comparably better ability to respond to and recover from the difficulties than their counterparts [79]. With the resilience framework, children who grow up with in poverty may exhibit positive outcomes [80]. Resilience provokes creative strategies to help people overcome poor life conditions [46]. For instance, they may reach out social support and community ties that help buffer the negative effects of economic distress. They also more likely to be involved in organized programs that assist with their needs and help them feel secure [81], which promotes resilience among children and youth. Hence, their capabilities and resilience can serve as a protective factor that attenuates the impact of cumulative family risks on their health. Nonetheless, up to date, not much is known about the epidemiology of childhood experiencing cumulative family risks among U.S. children through population-based studies [82]. Although resilience was not directly measured and analyzed in this study, the findings of this study suggest the potential role of resilience. Further research is warranted to investigate the role of resilience in the relation between cumulative family risk and child health especially in disadvantaged families. This finding also points to the importance of prevention efforts than interventions. Cultivating resilience and capabilities for children in adverse circumstances may be more efficient to promote child health in disadvantaged families than implementing interventions. In this manner, disadvantaged children may have better chances to pursue health equity and survive the inherited family risks.
